# Integration of transplanted chemically induced liver progenitor cells into recipient bile ducts in a rat bile duct transection model

**DOI:** 10.1371/journal.pone.0359343

**Published:** 2026-10-13

**Authors:** Peilin Li, Akihiko Soyama, Daisuke Miyamoto, Takanobu Hara, Hajime Matsushima, Ayaka Kinoshita, Kazushige Migita, Ayaka Satoh, Takashi Hamada, Hajime Imamura, Luyang Li, Tomohiko Adachi, Weili Gu, Susumu Eguchi

**Affiliations:** 1 Department of Surgery, Division of Digestive Surgery and Transplantology, Nagasaki University Graduate School of Biomedical Sciences, Nagasaki, Japan; 2 Department of Hepatobiliary Surgery II, Zhujiang Hospital, Southern Medical University, Guangzhou, China; 3 Guangdong Provincial Research Center for Artificial Organ and Tissue Engineering, Guangzhou Clinical Research and Transformation Center for Artificial Liver, Institute of Regenerative Medicine, Zhujiang Hospital, Southern Medical University, Guangzhou, China; 4 Department of Surgery, Guangzhou First People’s Hospital, School of Medicine, South China University of Technology, Guangzhou, Guangdong, China; King Faisal University, SAUDI ARABIA

## Abstract

**Background:**

Chemically induced liver progenitor cells (CLiPs) generated from mature hepatocytes are bipotent and represent a promising source for biliary repair. However, their therapeutic potential in cholangiopathy models remains unclear. In this study, we investigated rat CLiPs using a rat bile duct transection (BDT) model.

**Methods:**

Rat CLiPs were induced using the Y-27632/A-83–01/CHIR99021 regimen, resulting in loss of the hepatocyte marker ALB and gain of the progenitor markers CD90 and EpCAM. GFP-labeled CLiPs (1 × 10^7) were transplanted into Sprague–Dawley rats following BDT via portal vein infusion. On postoperative days (POD) 3 and 7, engraftment, proliferation, differentiation, and injury indices were assessed using immunofluorescence, quantitative reverse-transcription polymerase chain reaction (qRT-PCR), and serum biochemistry.

**Results:**

CLiPs engrafted and survived until POD 7 after portal vein infusion, and proliferative activity was observed by Ki-67 staining. Immunofluorescence revealed GFP + /ALB+ hepatocyte-like cells and GFP + /CK7 + cholangiocyte-like cells integrating near native ducts, indicating bipotential differentiation. qRT-PCR showed that BDT-induced expression of progenitor/ductular markers (Sox9, Epcam, Krt7, and Krt19) was attenuated in CLiP-treated groups compared to untreated BDT rats, although levels remained above those in sham rats. Serum AST, ALT, and ALP levels tended to decrease, and albumin levels increased after CLiP transplantation.

**Conclusions:**

CLiPs survived and acquired hepatocyte-like and cholangiocyte-like phenotypes during the early phase after portal vein transplantation in a rat BDT model. These findings support the feasibility of CLiP-based hepatobiliary regenerative therapy, although longer-term and functional studies are required to confirm durable biliary repair.

## 1. Introduction

Biliary diseases, including intrahepatic bile duct injury due to ischemia, present a significant clinical challenge owing to limited regenerative capacity of the biliary epithelium and lack of effective treatment strategies beyond surgical interventions or liver transplantation [1]. Damage to the intrahepatic bile ducts often leads to cholestasis, progressive liver fibrosis, and ultimately liver failure [2]. Consequently, there is a significant demand for regenerative strategies that can effectively repair or replace damaged bile ducts.

A major challenge is the scarcity of primary cholangiocytes; adult livers yield very few biliary epithelial cells for transplantation. In contrast, a large number of hepatocytes could be isolated. To exploit this, Katsuda et al. developed a small-molecule cocktail (Y-27632, A-83–01, CHIR99021) that reprograms mature hepatocytes into proliferative “chemically induced liver progenitor” (CLiP) cells [3]. These CLiPs are bipotent, maintain progenitor markers in culture, and can differentiate into hepatocytes and cholangiocytes. For instance, we have reported that aged rat hepatocytes can be converted into CLiPs with the capacity to mature into both hepatocytic and cholangiocyte lineages [4]. These findings suggest that CLiPs are an abundant source of biliary cells. Consistent with this, we also bioengineered three-dimensional tubular bile duct-like structures from rat CLiPs in vitro. When cocultured with hepatocytes, these CLiP-derived ducts were functional and supported bile transport analogs [5]. Collectively, these studies indicate that CLiPs have an intrinsic ability to generate bile duct-like tissues in vitro.

However, it has not been established whether CLiPs can engraft, survive, or form bile duct structures in vivo after biliary injury. Bile duct transection (BDT) in rodents, a common model in cholangiopathy research, induces obstructive cholestasis with marked proliferation of the biliary epithelium. In the present study, we examined rat CLiPs using a rat model of BDT.

Green Fluorescent Protein-positive (GFP)-labeled rat CLiPs were transplanted into Sprague-Dawley (SD) rats immediately after BDT using portal vein infusion. We assessed the survival, distribution, and differentiation of the transplanted cells. Here, we show that CLiPs engraft and survive in the injured liver and that many CLiP-derived cells acquire hepatocyte or cholangiocyte markers and integrate with native bile ducts, suggesting a role for CLiPs in bile duct repair.

## 2. Methods

### 2.1 Animals

Male GFP SD rats (SD-Tg(CAG-EGFP), SLC, Osaka, Japan) (GFP-SD rats) and Male SD rats (age: 7 weeks, 160–190 g; CLEA Japan Inc., Tokyo, Japan) were used in this study. The rats were bred and kept in conventional rat enclosures following a 12-hour cycle of light and darkness, with unrestricted access to both water and rat feed. All animal experiments adhered to the protocols approved by the Institutional Animal Care Committee of Nagasaki University and were conducted in accordance with the pertinent guidelines and regulations of Nagasaki University. This study was conducted in accordance with the ARRIVE guidelines. The Animal Research Ethics Committee of Nagasaki University approved the isolation and culturing of liver cells (approval number: 2106171722).

### 2.2 Rat hepatocyte isolation

Male GFP-SD rats (age: 7–8 weeks; weight: 160–200 g; CLEA Japan, Inc.) were used in this study. For primary rat mature hepatocyte (rMH) isolation, we employed a two-step collagenase perfusion method in accordance with previous reports [6,7]. Briefly, we cannulated the liver with an 18‐G cannula through the portal vein and perfused it with preperfusion buffer, followed by collagenase buffer. All buffer solutions were prewarmed to 37°C before isolation. We transferred the liver to a 100 mm sterile glass dish, minced the liver into pieces using surgical scissors, collected the hepatocyte suspension, and filtered it through a cotton mesh membrane and a 45 μm stainless steel mesh. The residual tissue in the cotton membrane was collected for biliary cell isolation. We then purified the filtered suspension containing hepatocytes thrice with isolation buffer by centrifugation at 50 g for 2 min each at 4°C, followed by centrifugation with 40% Percoll Plus solution (GE Healthcare) at 50 g for 20 min at 4°C. All experiments were performed using hepatocytes with at least 90% viability, as determined by the trypan blue exclusion test.

### 2.3 Rat CLiP induction

Rat CLiPs were generated from mature hepatocytes using a small-molecule cocktail as previously reported [3,6]. The rMH were seeded into collagen type-I-coated dishes (Asahi Techno Glass, Tokyo, Japan) at a density of 1 × 10^4^ cells/cm^2^ in small chemically reprogrammed culture medium of DMEM/F12 medium containing 2.4 g/L NaHCO3 and L-glutamine, supplemented with 5 mM HEPES, 30 µg/mL L-proline, 0.5 mg/mL BSA, 10 ng/mL epidermal growth factor, insulin-transferrin-serine-X, 0.1 µM dexamethasone, 10 mM nicotinamide, 1 mM ascorbic acid-2 phosphate, 100 U/mL penicillin, 100 mg/mL streptomycin, and containing two small chemical molecules of 0.5 μM A-83–01 (TGF-b type I receptor inhibitor, Wako Pure Chemical, Tokyo, Japan), 3 μM CHIR99021 (GSK-3 inhibitor, AdooQ BioScience, CA, USA), and 10 µM Y-27632, which would be called YAC medium [6,8]. The culture medium was changed 1 day after seeding and every 2–3 days thereafter. rCLiPs were typically generated from rMHs within 14–16 days and reached approximately 90% confluence.

### 2.4 Experimental protocol

The animals were randomly assigned to one of the following groups: normal control group (NOR; n = 5), BDT group (BDT; n = 10), and BDT plus portal vein CLiP transplantation group (PVI; n = 10). For animals assigned to POD 3 and POD 7 analyses, the number of rats used at each time point is indicated in the corresponding figure legends.

For performing a BDT in SD rats, the BDT could promote the significant proliferation of biliary epithelial cells and induce liver damage, which was used to study the potential differentiation capacity of biliary epithelial cells and the efficiency of rCLiPs in bile duct and liver injury and repair [9]. The study protocol was approved by the Research Center for Biomedical Models and Animal Welfare, Nagasaki University Graduate School of Biomedical Sciences (approval number: 2106171722). All rats were anesthetized with isoflurane during invasive procedures.

For CLiPs transplantation in the PVI group, rCLiPs at 90–100% confluence were treated with TrypLE Express for 15–20 min. After live cell counting using trypan blue, 1 × 10^7 viable cells were suspended in 1 mL PBS and infused into rats immediately after BDT via the portal vein using a temporary clamp. The abdominal cavity was closed after confirmation of hemostasis and rinsing.

Animals were monitored daily, and twice daily during the postoperative period or if clinical abnormalities appeared. Humane endpoints were predefined. Rats were euthanized if any of the following criteria were met: ≥ 20% body weight loss from baseline; Severe lethargy, inability to obtain food/water, or persistent lack of grooming; Signs of respiratory distress, severe dehydration, or continuous abdominal pain (hunched posture, guarding); Infection, ulceration, or necrosis at surgical or injection sites; Any condition determined by veterinary staff to cause unrelievable pain or distress. Rats meeting humane endpoint criteria were euthanized immediately using CO_2_ inhalation followed by cervical dislocation. No animals died unexpectedly prior to meeting humane endpoint criteria. No rats met the predefined exclusion criteria, and all animals assigned to each group were included in the final analyses.

### 2.5 Liver histology

The rats were sacrificed under anesthesia on day 7, and liver tissues were collected immediately. Liver tissue was fixed with 4% paraformaldehyde in phosphate-buffered saline (PBS; Wako Pure Chemical, Osaka, Japan) for 3 days. Fixed tissues were embedded in paraffin, cut into 5-μm sections, and deparaffinized.

For immunohistochemical staining, tissue sections of the liver tissue were stained for Ki-67 and GFP. Staining procedures were performed according to the recommended protocols.

Cells were subjected to immunofluorescence staining using antibodies against albumin (ALB) Epcam and CD90, and the nuclei were counterstained with 4′,6-diamidiono-2-phenylindole (DAPI; DOJINDO, Kumamoto, Japan).

### 2.6 Gene expression analysis by quantitative reverse-transcription polymerase chain reaction (QRT-PCR)

Total mRNA of the liver tissues was extracted using a spin column (NucleoSpin RNA II; Macherey-Nagel, Düren, Germany). cDNA was synthesized using a high-capacity cDNA reverse transcription kit (Applied Biosystems, Tokyo, Japan). Samples were then stored at −20°C until their analysis by polymerase chain reaction (PCR), which was performed using an Applied Biosystems StepOne Plus Real-time PCR System with TaqMan Gene Expression Assay Kits (Applied Biosystems, Tokyo, Japan) according to the manufacturer’s instructions. Briefly, PCR mixtures contained 1 μL of cDNA, 1 μL of TaqMan Gene Expression Assay probe, 5 μL of TaqMan Fast Advanced Master Mix (both from Applied Biosystems), and 13 μL of nuclease-free water. The thermocycling conditions were 95°C for 20 s, followed by 40 cycles of 95°C for 1 s and 60°C for 20 s. Expression levels were quantified using the comparative cycle time method. Cycle threshold (Ct) values were automatically determined by the Applied Biosystems StepOne Plus Real-Time PCR System, and fold changes in gene expression were calculated by the 2^(−ΔΔCT) method. Expression levels were normalized to those of the housekeeping gene and internal control glyceraldehyde 3-phosphate dehydrogenase. The original information was attached in the Supporting Information sheet S2 Table.

### 2.7 Immunofluorescence staining

The liver sections and the rCLiPs were fixed with 4% paraformaldehyde in PBS (Wako Pure Chemical, Osaka, Japan) for 10 min. The fixed samples were incubated in 0.1% Triton X-100 (Sigma-Aldrich, Tokyo, Japan) in PBS for 10 min and blocked in PBS containing 1% BSA for 1 hour at room temperature. The sections were then incubated with primary antibodies diluted in PBS + 1% BSA at 4°C overnight. After washing with PBS thrice, the membranes were incubated with the appropriate secondary antibodies diluted in PBS + 1% BSA for 2 hours. All primary and secondary antibodies are listed. Nuclei were stained with DAPI (DOJINDO, Kumamoto, Japan) for 30 min. The cells were then washed thrice with PBS for 30–60 min. Fluorescence and bright-field images were captured using a microscope (Ti-U and C-HGFI; Nikon, Tokyo, Japan).

### 2.8 Statistical analysis

Statistical analyses were performed using GraphPad Prism 8.0. (GraphPad Software Inc., San Diego, CA, USA). Data are presented as mean ± SEM. For comparisons among the NOR, BDT, and PVI groups, one-way ANOVA followed by Tukey’s multiple-comparison test was used. When only two groups were compared, an unpaired two-tailed Student’s t-test was used. Exact p-values are shown in the corresponding figures or figure legends. A value of p < 0.05 was considered statistically significant.

## 3. Results

### 3.1 Rat GFP-CLiPs were induced from isolated mature hepatocytes

Rat CLiPs were induced using a small chemical molecule culture medium as previously reported [3,6,10]. Mature hepatocytes were cultured in YAC medium for 14 days and subsequently induced to dedifferentiate into liver progenitor-like cells. During induction, mature hepatocytes gradually altered their multipolarized phenotype, exhibited an increased nuclear-to-cytoplasmic ratio, acquired proliferative capacity, and maintained their cellular morphology in subsequent passages (Fig 1A and 1B). Further phenotypic characterization using immunofluorescence staining revealed that these transformed cells expressed liver progenitor markers, including CD90 and EpCAM, but did not express the mature hepatocyte protein ALB (Fig 1C). These results indicate that GFP-positive mature hepatocytes were successfully induced into liver progenitor cells that stably expressed the GFP marker.

**Fig 1:**
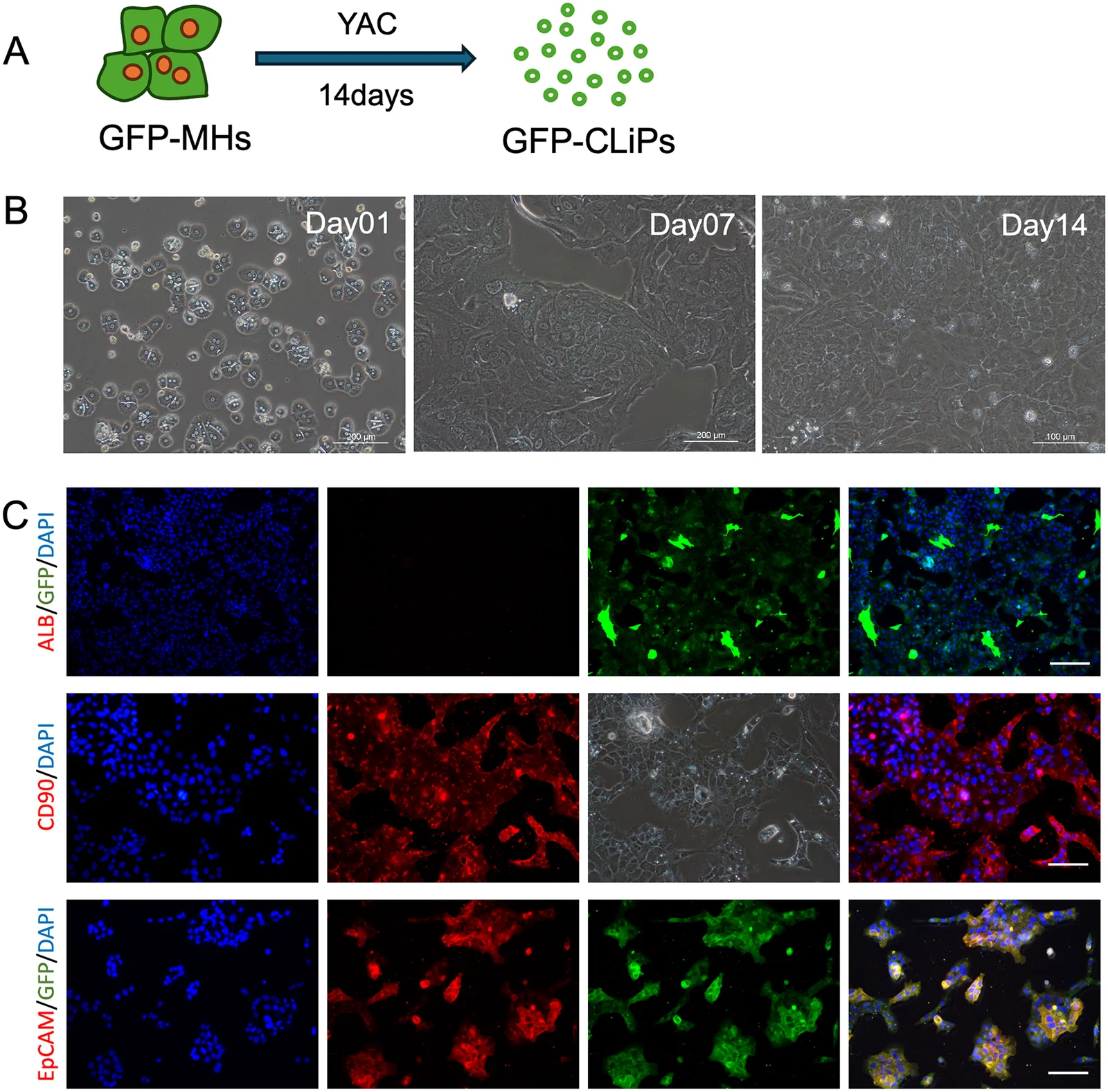
Rat GFP-CLiP were induced from isolated mature hepatocytes. A. Rat GFP-CLiP were induced from isolated mature hepatocytes by YAC medium. B. The morphology of the cells changed into small cells with the capacity for proliferation during the culturing in FAC medium (a small molecule combination medium). C. The immunofluorescent staining results showed that the GFP-CLiP expressed the liver progenitor cell markers of CD90 and EpCAM and was negative for mature hepatocyte ALB marker. Scale bar = 100 μm. At least three independent experiments were conducted, and the representative images are shown in this figure.

### 3.2 Early survival and proliferative activity of transplanted GFP-CLiPs after BDT

To investigate the role of CLiPs in cholangiopathy, an experimental BDT model was established using SD rats (Fig 2A). Following BDT surgery, 1 × 10^7 GFP-labeled CLiPs suspended in 1 mL PBS were infused into the liver via the portal vein. Rats were euthanized on POD 3 and POD 7 to assess the survival and distribution of transplanted cells (Fig 2A). GFP-positive cells were detected at both time points, indicating early survival of transplanted CLiPs (Fig 2B). Ki-67 immunostaining was performed to assess proliferative activity after transplantation. Compared with POD 3, POD 7 showed increased Ki-67-positive cells in regions containing GFP-positive transplanted cells, suggesting an active proliferative response during the early phase after CLiP transplantation (Fig 2C). Because quantitative lineage-specific proliferation analysis was not performed, these findings just provided evidence of engraftment-associated proliferative activity rather than definitive proof of clonal expansion of transplanted cells.

**Fig 2:**
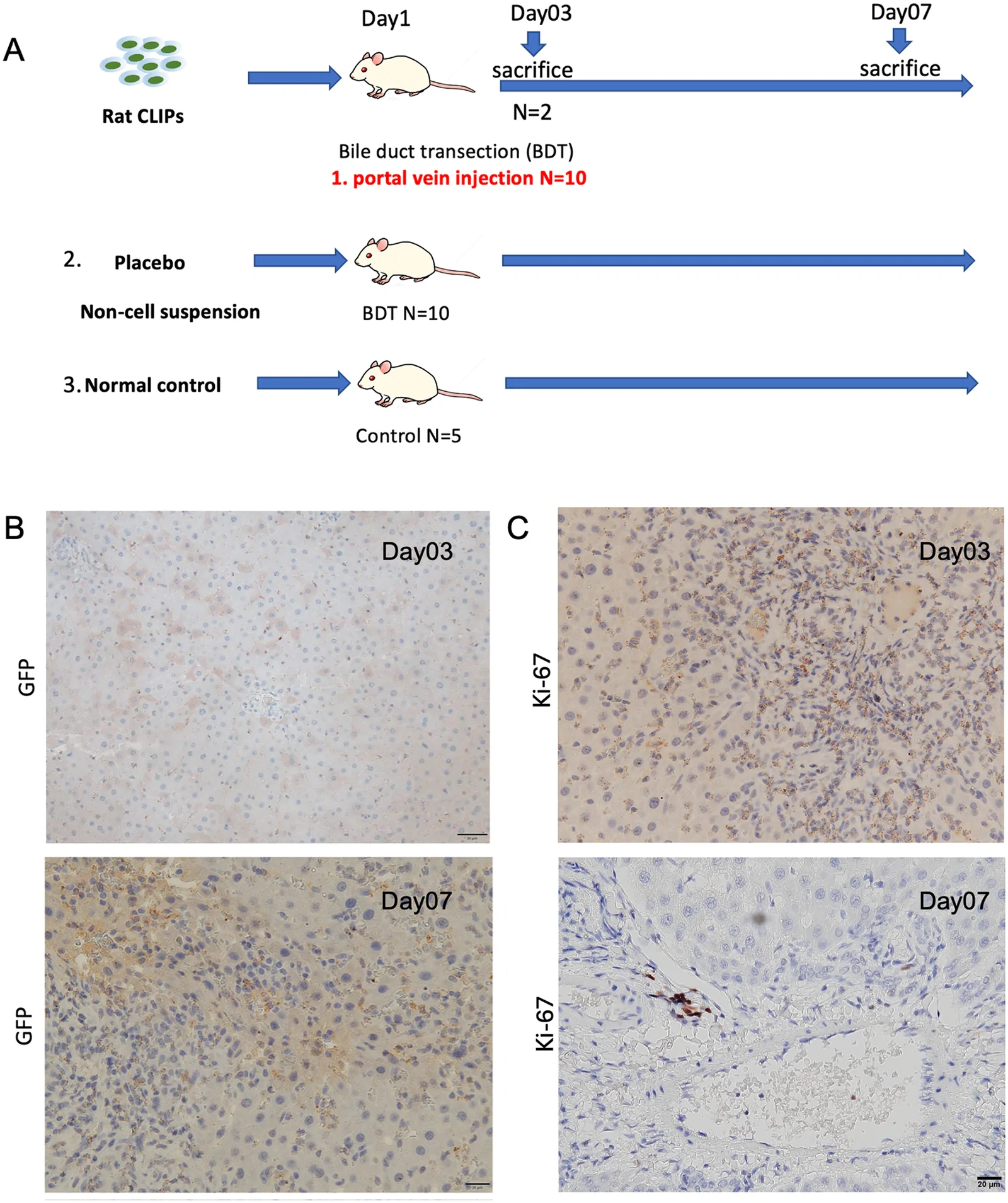
GFP-CLiP could survive in vivo in the liver (post-operation day 7 (POD 7)) after the common bile duct transection. A. Schematic overview of the BDT model and portal vein infusion of GFP-labeled CLiPs, followed by tissue collection on POD 3 and POD 7. B. Immunohistochemistry staining results showed that GFP-CLiP could survive in the liver on POD 3 and 7. Scale bar = 50 μm (B) and 20 μm (C). C. Ki-67 immunostaining showed increased proliferative activity on POD 7 compared with POD 3 in regions containing GFP-positive transplanted cells. Scale bar = 20 μm. N = 3–6. At least three independent experiments were conducted, and the representative images were shown in this figure.

### 3.3 GFP-CLiPs acquired hepatocyte-like and cholangiocyte-like phenotypes after portal vein transplantation

To evaluate the in vivo differentiation potential of CLiPs and their role in bile duct repair in a BDT model, we transplanted GFP-labeled CLiPs into the livers of SD rats via portal vein injection. On day 7 post-transplantation, the animals were euthanized for tissue collection. Immunofluorescence analysis revealed the presence of GFP/ALB double-positive cells in the PVI group, suggesting that a subset of transplanted CLiPs acquired a hepatocyte-like phenotype in vivo. Moreover, cells co-expressing CK-7 and GFP were observed within the native bile ducts, suggesting that transplanted CLiPs acquired a cholangiocyte-like phenotype and were structurally associated with native bile duct regions (Fig 3A).

**Fig 3:**
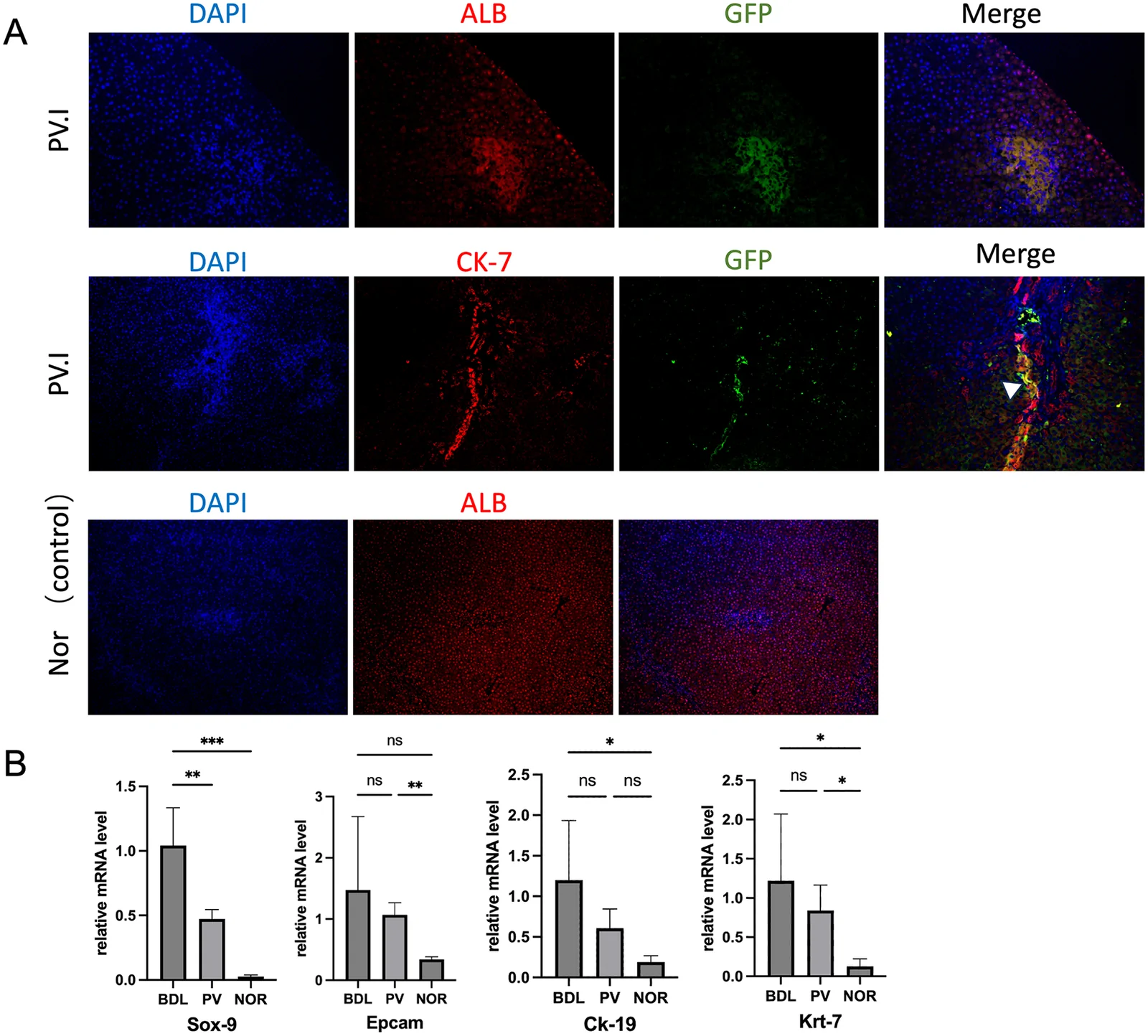
GFP-CLiP had the biopotential capacity of differentiation of biliary epithelial cells and mature hepatocytes in the liver on POD 7. A. The immunofluorescent staining results showed that there were cells with double positive of GFP and ALB and double positive of GFP and CK-7. Scale bar = 100 μm. The white arrow showed that the cell with double staining of CK-7 and EpCAM combinate into the regenerative bile duct or native bile duct. B. The qRT-PCR results showed that qRT-PCR analysis showed that portal vein CLiP transplantation partially reduced the BDT-induced mRNA expression of Sox9, Epcam, Krt19, and Krt7. Values were determined relative to GAPDH and presented as fold-change relative to the control group. One-way ANOVA: ns > 0.05, *p < 0.05, **p < 0.01, ***p < 0.001.

Furthermore, quantitative RT-PCR analysis of liver tissues 7 days post-transplantation showed that the expression levels of Sox9, Epcam, Ck19, and Krt7 were lower in the PVI group than in the BDT group but remained higher than those in the normal control group (Fig 3B and S2 Table). These results suggest that CLiPs attenuate bile duct reactions typically induced by BDT.

Finally, ELISA analysis of plasma liver injury markers revealed that BDT leads to a significant increase in the hepatic injury markers aspartate aminotransferase (AST) and alanine aminotransferase (ALT), as well as the biliary injury marker alkaline phosphatase (ALP), accompanied by a decrease in ALB, a marker of liver function (Fig 4A–4D). Although CLiP transplantation showed a trend toward hepatoprotection, the differences were not statistically significant. The original information of plasma was attached in the Supporting Information S1 Table (S1 Table).

**Fig 4:**
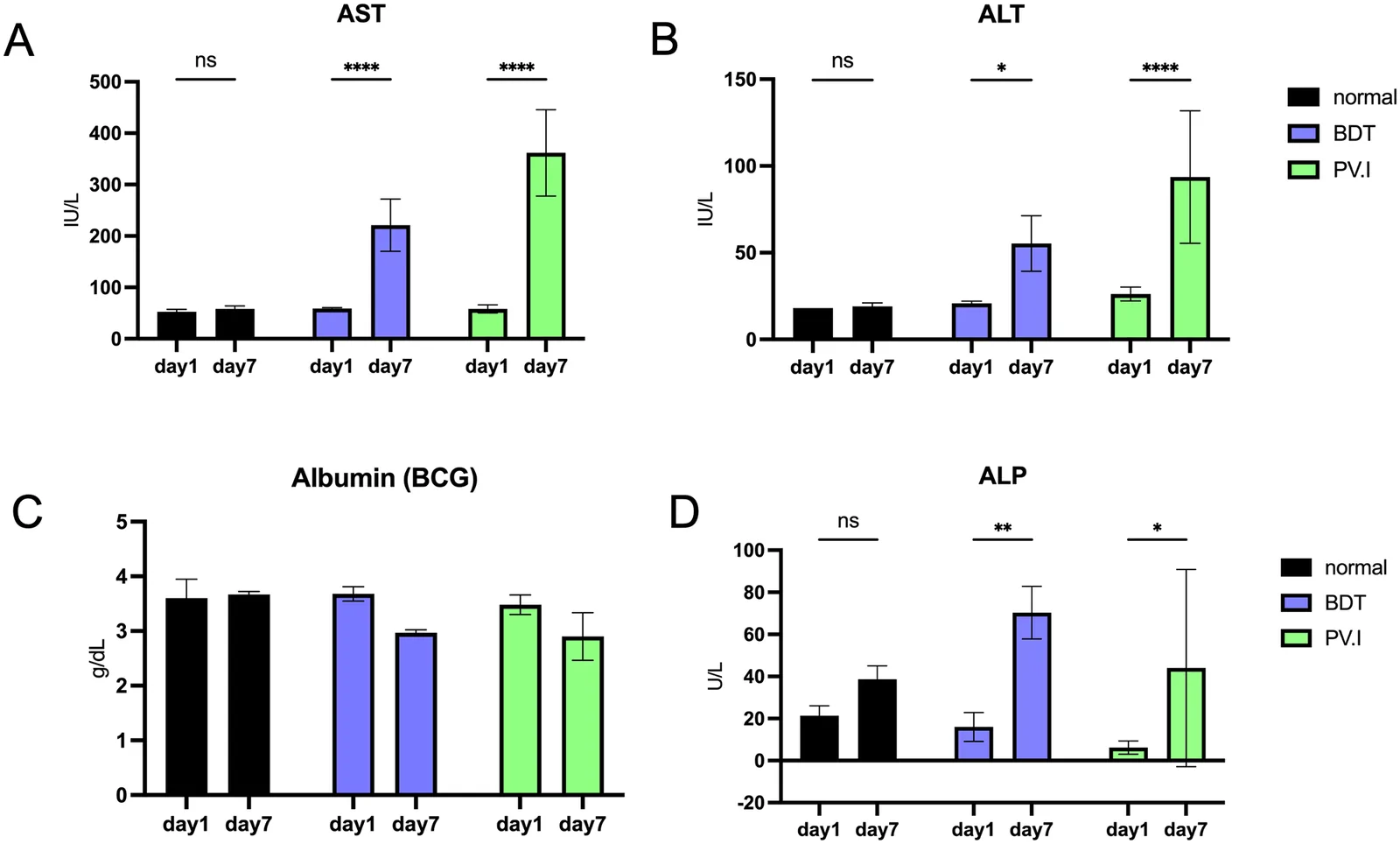
Effects of portal vein CLiP transplantation on serum liver injury markers after BDT. Serum levels of AST (A), ALT (B), albumin (C), and ALP (D) were measured on POD 7. BDT increased serum AST, ALT, and ALP levels and decreased albumin compared with the normal control group. Portal vein CLiP transplantation showed a trend toward improvement in serum injury markers, although most differences between the BDT and PVI groups did not reach statistical significance. Data are presented as mean ± SEM. Statistical analysis was performed using one-way ANOVA followed by multiple-comparison testing. ns > 0.05, *p < 0.05, **p < 0.01, ***p < 0.001.

## 4. Discussion

This study provides early proof-of-concept evidence that CLiPs derived from mature hepatocytes can survive after portal vein transplantation into a rat BDT model and acquire hepatocyte-like and cholangiocyte-like phenotypes in vivo. Our findings support the feasibility of CLiP-based therapy as a potential approach to address the biliary damage associated with cholangiopathies.

Our findings demonstrate, for the first time, that transplanted CLiPs can engraft into the injured biliary tract and contribute to bile duct structures in vivo. One-week post-transplantation, GFP-positive CLiP-derived cells were readily detected in the livers of treated rats, and a subset of these cells co-expressed lineage markers. Specifically, some GFP-positive cells were ALB-positive, indicating differentiation into mature hepatocytes, whereas others co-expressed GFP and cytokeratin-7, a cholangiocyte marker, within the native bile duct regions. The presence of GFP + /CK7 + cells closely associated with bile duct walls strongly suggests that CLiPs differentiate into biliary epithelial cells and integrate into the existing ducts. This bipotential differentiation in vivo is consistent with previous reports that CLiPs have a dual lineage capacity. Katsuda et al. originally showed that chemically reprogrammed hepatocytes could become either hepatocytes or BECs [3], and our group later confirmed that CLiPs from aged hepatocytes mature into cholangiocytes. Our results extend these in vitro observations by demonstrating that CLiPs can have both fates in a living liver [4].

Integration of CLiPs into ductal structures mirrors in vitro bioengineering studies. We have previously reported engineered CLiP-derived tubular biliary structures that support bile transport when cultured with hepatocytes [5].

Likewise, in this study, transplanted CLiPs not only survived but also apparently assembled into bile duct-like architectures in situ. Although we did not directly measure bile flow or transport, the anatomical localization and marker expression of CLiP-derived cells suggest structural association with native ductal regions, although functional incorporation remains to be demonstrated. Furthermore, CLiP-treated livers showed a blunted ductular reaction: expression of progenitor/ductal genes (Sox9, CK19, and Krt7) was elevated by BDT but was somewhat reduced in CLiP-injected groups compared to that in untreated BDT. This suggests that CLiPs attenuate the proliferative ductular response to injury, possibly by contributing to the functioning of the biliary epithelium. The ductular reaction is a marker of pathological repair [11]. In bile duct injury caused by ischemia or antibody-mediated rejection after liver transplantation, the ductular reaction progresses as a regenerative response, owing to the inability of damaged bile ducts to regenerate. Normally, when the bile duct epithelium is injured, repair occurs through the activation of surrounding bile duct progenitor cells or ductularization of hepatocytes. However, in cases of severe injury due to ischemic bile duct damage or rejection, regeneration of the existing bile ducts is insufficient, and as a compensatory response, the ductular reaction in the portal area is upregulated [1]. Although the ductular reaction is a phenomenon that attempts to facilitate repair, if excessive or persistent, it leads to fibrosis progression [12]. In this study, CLiP transplantation via the portal vein was associated with a trend toward reduced ductular reaction after BDT, suggesting that transplanted CLiPs may partially attenuate injury-induced biliary responses.

A similar phenomenon was observed following CLiP transplantation in other injury models in our previous reports. For example, rat CLiPs delivered to a murine NASH model home to the liver significantly reduced fibrosis and injury markers [13]. Analogously, our CLiP-treated rats trended towards lower serum AST, ALT, and ALP levels and higher ALB levels than the untreated BDT rats, although these differences did not reach statistical significance in our small cohort.

Despite these encouraging findings, our study has limitations. First, the observation period was limited to POD 7; therefore, the durability of CLiP engraftment, long-term differentiation, and potential adverse effects such as aberrant proliferation or fibrosis remain unknown. Second, although GFP/CK7-positive cells were observed near native bile ducts, the present study did not include lineage-tracing approaches, three-dimensional ductal reconstruction, or direct functional assays such as bile flow measurement or bile acid transport assessment. Therefore, true functional integration into the biliary system remains to be confirmed. Third, the sample size was relatively small, and some biochemical changes did not reach statistical significance. Finally, a route-matched vehicle-only portal infusion group was not included, and potential effects related to the portal injection procedure itself could not be completely excluded. Future studies should further optimize the delivery strategy, cell dose, and timing of CLiP transplantation to improve engraftment efficiency and functional biliary repair. It is also important to examine whether CLiPs induce any adverse effects, such as aberrant proliferation or fibrosis, although none were evident in our 7-day data.

In conclusion, our results provide proof-of-concept evidence that portal vein-transplanted CLiPs can survive in the injured liver and acquire hepatocyte-like and cholangiocyte-like phenotypes during the early phase after BDT. These findings support the potential of CLiP-based hepatobiliary regenerative therapy, although longer-term studies and functional validation are required to confirm durable biliary repair. Future studies in larger animals and eventual clinical translation are needed to fully understand the therapeutic potential of CLiPs in biliary diseases.

## Supporting information

S1 Table Result of plasma levels. (XLSX)

S2 Table Original results of qPCR. (XLSX)

## Abbreviations

CLiP: Chemically induced liver progenitor
BDT: Bile duct transection
GFP: Green Fluorescent Protein-positive
SD: Sprague-Dawley
rMH: Rat mature hepatocyte
rCLiPs: Rat CLiPs
qRT-PCR: Quantitative reverse-transcription polymerase chain reaction
PCR: Polymerase chain reaction
Ct: Cycle threshold
PBS: Phosphate-buffered saline
ALB: Albumin
AST: Aspartate aminotransferase
ALT: Alanine aminotransferase
ALP: Alkaline phosphatase
